# Genome sequence of *Helicobacter suis *supports its role in gastric pathology

**DOI:** 10.1186/1297-9716-42-51

**Published:** 2011-03-17

**Authors:** Miet Vermoote, Tom Theo Marie Vandekerckhove, Bram Flahou, Frank Pasmans, Annemieke Smet, Dominic De Groote, Wim Van Criekinge, Richard Ducatelle, Freddy Haesebrouck

**Affiliations:** 1 Department of Pathology, Bacteriology and Avian Diseases, Faculty of Veterinary Medicine, Ghent University, Merelbeke, Belgium; 2 Laboratory for Bioinformatics and Computational Genomics, Department of Molecular Biotechnology, Faculty of Bioscience Engineering, Ghent University, Ghent, Belgium; 3 Department of Pharmacology, Faculty of Pharmaceutical Sciences, Ghent University, Ghent, Belgium

## Abstract

*Helicobacter *(*H.*) *suis *has been associated with chronic gastritis and ulcers of the pars oesophagea in pigs, and with gastritis, peptic ulcer disease and gastric mucosa-associated lymphoid tissue lymphoma in humans. In order to obtain better insight into the genes involved in pathogenicity and in the specific adaptation to the gastric environment of *H. suis*, a genome analysis was performed of two *H. suis *strains isolated from the gastric mucosa of swine. Homologs of the vast majority of genes shown to be important for gastric colonization of the human pathogen *H. pylori *were detected in the *H. suis *genome. *H. suis *encodes several putative outer membrane proteins, of which two similar to the *H. pylori *adhesins HpaA and HorB. *H. suis *harbours an almost complete *comB *type IV secretion system and members of the type IV secretion system 3, but lacks most of the genes present in the *cag *pathogenicity island of *H. pylori*. Homologs of genes encoding the *H. pylori *neutrophil-activating protein and γ-glutamyl transpeptidase were identified in *H. suis*. *H. suis *also possesses several other presumptive virulence-associated genes, including homologs for *mviN*, the *H. pylori *flavodoxin gene, and a homolog of the *H. pylori *vacuolating cytotoxin A gene. It was concluded that although genes coding for some important virulence factors in *H. pylori*, such as the cytotoxin-associated protein (CagA), are not detected in the *H. suis *genome, homologs of other genes associated with colonization and virulence of *H. pylori *and other bacteria are present.

## Introduction

*Helicobacter *(*H.*) *suis *is a very fastidious, spiral-shaped, Gram-negative bacterium requiring a biphasic culture medium at pH 5 enriched with fetal calf serum, and a microaerobic atmosphere for in vitro growth [1]. *H. suis *colonizes the stomach of more than 60% of slaughter pigs [1,2]. Although the exact role of *H. suis *in gastric disease in pigs is still unclear, it has been associated with chronic gastritis [3,4] and ulcers of the pars oesophagea of the stomach [5-7]. This may result in significant economic losses due to sudden death, decreased feed intake and reduced daily weight gain [8]. A reduction of approximately 20 g/day in weight gain was observed in animals experimentally infected with *H. suis*, compared to the non-infected control animals [9].

Bacterial gastric disorders in humans are mainly caused by *Helicobacter pylori *[10]. However, non-*Helicobacter pylori *helicobacters (NHPH) have also been associated with human gastric disease with a prevalence ranging between 0.2 and 6% [5]. *H. suis *is the most frequent NHPH species found in humans, where it was originally named "*H. heilmannii*" type 1 [11]. There are strong indications that pigs may serve as a source of infection for humans [5,12]. In the human host, *H. suis *has been associated with peptic ulcer disease [13], gastric mucosa-associated lymphoid tissue (MALT) lymphoma [14] and chronic gastritis [15]. In rodent models of human gastric disease, the bacterium causes severe inflammation and MALT lymphoma-like lesions [16].

Up to now, little is known about the pathogenesis of *H. suis *infections. To improve understanding in the genes playing a role in pathogenicity, gastric colonization and persistence of *H. suis*, a genome-wide comparison with the well-investigated *H. pylori *genome was performed. Some virulence factors may indeed be similar for both bacteria. As there may also be differences, ab initio annotations of the *H. suis *genome were performed as well.

## Materials and methods

### Genome sequencing

A pyrosequencing (454 Life Sciences Corporation, Branford, CT, USA) assay was applied to the genome of the type strain of *H. suis *(HS1^T ^= LMG 23995^T ^= DSM 19735^T^) and *H. suis *strain 5 (HS5), isolated from the gastric mucosa of two different swine, according to the method described by Baele et al. [1]. Quality filtered sequences were assembled into contigs using a 454 Newbler assembler (Roche, Branford, CT, USA).

### Functional annotation

In order to maximize the number of quality gene annotations, two different annotating approaches were followed: cross-mapping with three *Helicobacter pylori *strains (26695, Shi470, and G27 with NCBI accession numbers NC_000915, NC_010698, and NC_011333, respectively), and ab initio annotation.

#### Cross-mapping annotation

A custom BLAST [17] database was created from the HS1^T ^and HS5 genomic contigs. The *H. pylori *proteome and non-coding RNAs were aligned (tblastn program of the BLAST suite, e threshold set to 10^-3^) to the *H. suis *database. For each BLAST hit the following additional information was analysed: 1) (secretion) signal peptide cleavage site if present, as assessed by the SignalP 3.0 program [18,19]; 2) specifications of transmembrane helices (number, start and end positions, presumed topology with regard to the cytoplasmic membrane) if present, as assessed by the TMHMM program [20]; 3) an estimate of the ribosome binding strength of the mRNA region preceding the most probable start codon. Ribosome binding strength was estimated by applying two established facts: i) on an mRNA strand, usually within 20 nucleotides before the actual start codon, the reverse complement of 5 to 7 nucleotides near the 16S rRNA 3' end acts as an attractor and positioner for the ribosomal small subunit; this region is known as the Shine-Dalgarno sequence [21,22]; ii) in Gram-negative bacteria an AU-rich mRNA region some 16 nucleotides long and immediately preceding the Shine-Dalgarno sequence may also attract and position ribosomes to help initiate translation of the correct, biologically active gene product [23,24]. For *H. suis*, the Shine-Dalgarno sequence was determined to be a subsequence of AGGAGGU (which is the reverse complement of the 3' end of the 16S rRNA), and the minimum AU-richness (equivalent to ribosome binding capacity) of the preceding region was arbitrarily set to 10/16. For each theoretical ORF a range of possible start codons was scored; the higher the similarity to the ideal Shine-Dalgarno sequence, or the AU-richer the preceding region, or the better a combination of both, the more likely the potential start codon is to be the actual start codon.

#### Ab initio annotation

For ab initio annotation, theoretical open reading frames (ORFs) were first determined using the EMBOSS getorf tool (with minimum ORF length set to 90 nucleotides, and taking all alternative start codons into account) [25]. All ORFs were translated subsequently, and BLAST (blastp program) was performed with an e threshold of 10^-15 ^against the Uniprot-KB universal protein database. The generalist algorithm of getorf yielded roughly a tenfold of the expected natural ORFs, reducing the risk of false negatives. In order to keep the false positive rate low, extra parameters were considered: 1) percentage alignment between query and hit ORFs; 2) percentage similarity or conservation between aligned portions of query and hit ORFs; 3) ribosome binding strength (for more details see above). To determine the presence of one or more conserved domains a rpsblast search (with default parameter values) was carried out for every single theoretical ORF against the compiled Conserved Domain Database which holds protein domain alignments from several other database sources [26].

## Results

### General features of the *H. suis *genome

In the HS1^T ^genome a total of 1 635 292 base pairs and in the HS5 genome 1 669 960 bp were sequenced, both with an average GC content of 40%. In contrast to *H. pylori*, only one copy of both the 16S and 23S rRNA genes was detected, but like *H. pylori*, *H. suis *has three copies of the 5S rRNA gene. Thirty-eight transfer RNAs were identified. On the whole, 1266 ORFs from HS1^T ^and 1257 from HS5 were detected, of which 194 and 191 encoded hypothetical proteins respectively. In 98 and 92 ORFs a signal peptide cleavage site was detected, demonstrating predicted secreted proteins of HS1^T ^and HS5 respectively. The TMHMM program predicted 210 and 206 proteins with at least one transmembrane helix for HS1^T ^and HS5 respectively. The sequence fraction identical for HS1^T ^and HS5 is henceforward described together as the "*H. suis *genome".

### Genes possibly involved in gastric colonization and persistence

Homologs of *H. pylori *genes involved in acid acclimation, chemotaxis, adhesion to gastric epithelial cells, oxidative stress resistance (Table 1), and motility were detected in the *H. suis *genome. The latter were identified as a flagellar biosystem similar to that of *H. pylori *[27]. Moreover, *H. suis *contains a fibrinonectin/fibrinogen-binding protein coding gene, but the corresponding protein lacks a transmembrane helix or signal peptide cleavage site according to the bioinformatics tools mentioned earlier. Homologs coding for CMP-N-acetylneuraminic acid synthetase (NeuA) (HSUHS1_0474, HSUHS5_0481), sialic acid synthase (NeuB) (HSUHS1_0477, HSUHS5_0478), and UDP-N-acetylglucosamine-2-epimerase (WecB) (HSUHS1_1107, HSUHS5_0784) were observed as well.

**Table 1:** Genes associated with pH homeostasis, chemotaxis, adhesion to epithelial cells, and oxidative stress resistance in the genome of *H. suis *type strain 1 (HS1^T^) and *H. suis *strain 5 (HS5).

Group	Gene detected in HS1^T^	Gene detected in HS5	Description of homolog	Percentage of sequence aligned (of which % conserved) with described homolog^1^
**pH homeostasis**	HSUHS1_0708	HSUHS5_0286	Urease subunit alfa (*ureA*) of *H. heilmannii*	100 (94)
	HSUHS1_0707	HSUHS5_0285	Urease subunit beta (*ureB*) of *H. heilmannii*	100 (94)
	HSUHS1_0706	HSUHS5_0284	Urease transporter (*ureI*) of *H. felis*	100 (89)
	HSUHS1_0705	HSUHS5_0283	Urease accessory protein (*ureE*) of *H. bizzozeronnii*	100 (84)
	HSUHS1_0704	HSUHS5_0282	Urease accessory protein (*ureF*) of *H. bizzozeronnii*	100 (84)
	HSUHS1_0702	HSUHS5_0280	Urease accessory protein (*ureH*) of *H. bizzozeronnii*	96 (84)
	HSUHS1_0703	HSUHS5_0281	Urease accessory protein (*ureG*) of *H. bizzozeronnii*	100 (95)
	HSUHS1_0133	HSUHS5_0547	Hydrogenase expression/formation protein (*hypA*) of *H. pylori*	98 (83)
	HSUHS1_0615	HSUHS5_0817	Hydrogenase expression/formation protein (*hypB*) of *H. pylori*	99 (91)
	HSUHS1_0616	HSUHS5_0816	Hydrogenase expression/formation protein (*hypC*) of *H. pylori*	98 (89)
	HSUHS1_0617	HSUHS5_0815	Hydrogenase expression/formation protein (*hypD*) of *H. achinonychis*	98 (80)
	HSUHS1_0081	HSUHS5_1197	l-Asparaginase II (*ansB*) of *H. pylori*	98 (64)
	HSUHS1_0230	HSUHS5_1130	Arginase (*rocF*) of *H. pylori*	99 (75)
	HSUHS1_0888	HSUHS5_0231	Acylamide amidohydrolase (*amiE*) of *H. pylori*	100 (93)
	HSUHS1_0680	HSUHS5_0265	Formamidase (*amiF*) of *H. pylori*	100 (98)
	HSUHS1_0161	HSUHS5_1077	α-Carbonic anhydrase of *H. pylori*	92 (69)
	HSUHS1_0391	HSUHS5_0874	Aspartase (*aspA*) of *H. acinonychis*	100 (89)

**Chemotaxis**	HSUHS1_1004	HSUHS5_0649	CheA-MCP interaction modulator of *H. pylori*	99 (79)
	HSUHS1_1003	-	Bifunctional chemotaxis protein (*cheF*) of *H. pylori*	82 (86)
	HSUHS1_1002	HSUHS5_0775	Purine-binding chemotaxis portein (*cheW*) of *H. pylori*	98 (91)
	HSUHS1_0538	HSUHS5_0706	Chemotaxis protein (*cheV*) of *H. pylori*	100 (92)
	HSUHS1_0846	HSUHS5_0081	Putative chemotaxis protein of *H. pylori*	100 (79)
	HSUHS1_0299	HSUHS5_0250	Chemotaxis protein (*cheY*) of *H. pylori*	100 (95)
	HSUHS1_1001	HSUHS5_0774	Methyl-accepting chemotaxis protein (*tlpA*) of *H. pylori*	100 (60)
	HSUHS1_0286	HSUHS5_0256	Methyl-accepting chemotaxis protein ( *tlpB*) of *H. pylori*	98 (63)
	HSUHS1_0479	HSUHS5_0476	Methyl- accepting chemotaxis protein of *H. acinonychis*	100 (66)
	HSUHS1_0196	HSUHS5_0122	Methyl- accepting chemotaxis protein of *Campylobacter upsaliensis *^2^	99 (53)
	HSUHS1_0141	HSUHS5_0641	Methyl- accepting chemotaxis protein of *Campylobacter fetus subsp. fetus *^2^	99 (64)
	HSUHS1_0763	-	Methyl- accepting chemotaxis protein of *Methylibium petroleiphilum *^*2*^	83 (52)
	HSUHS1_0944	HSUHS5_0990	Methyl-accepting chemotaxis sensory transducer *Marinomonas sp.*^2^	57 (59)

**Adhesion**	HSUHS1_0666	HSUHS5_1053	Outer membrane protein (*horB*) of *H. pylori*	100 (63)
	HSUHS1_0354	HSUHS5_0398	Neuraminyllactose-binding hemagglutinin *(hpaA) *of *H. acinonychis*	94 (77)

**Oxidative stress resistance**	HSUHS1_1147	HSUHS5_0608	Catalase (*katA*) of *H. acinonychis*	95 (82)
	HSUHS1_0549	HSUHS5_1206	Mismatch repair ATPase (*mutS*) of *H. hepaticus*	99 (60)
	HSUHS1_0163	HSUHS5_0495	Superoxide dismutase (*sodB*) of *H. pylori*	100 (90)
	HSUHS1_1186	HSUHS5_0005	Bacterioferritin co-migratory protein of *H. hepaticus*	99 (72)
	HSUHS1_0683	HSUHS5_0262	NAD(P)H quinone reductase (*mdaB*) of *Campylobacter fetus subsp. fetus*	97 (68)
	HSUHS1_0689	HSUHS5_0268	Peroxiredoxin of *H. pylori *^3^	100 (92)

^1 ^Resulting from tblastn-based cross-mapping of the *H. pylori *proteome to the *H. suis *HS1^T ^and HS5 genomes and blastp-based *ab initio *analyses of the translated *H. suis *HS1^T ^and HS5 ORFs against the Uniprot-KB universal protein database. Differences between HS1^T ^and HS5 homologs ≤ 1%.

^2 ^Lacking in other *Helicobacter *genomes available at GenBank.

^3 ^Member of the 2-Cys peroxiredoxin superfamily.

Genes encoding putative outer membrane proteins (OMPs) in relation to *H. pylori *OMPs are presented in Additional file 1 Table S1. Genes coding for members of major *H. pylori *OMP families (Hop, Hor, Hof proteins, iron-regulated and efflux pump OMPs) could be aligned with the *H. pylori *genome. Both *H. suis *strains contain the *hof *genes *hofA*, *C*, *E*, *F*, the *hop *genes *hopE*, *G-2 *and *H*, and the *hor *genes *horB*, *C*, *D*, and *J*. Additionally, HS1^T ^contains homologs of the *hopW *protein precursor and *horE*, whereas HS5 possesses additional homologs of *horA*, *horF*, and *horL*. No members of the *Helicobacter *outer membrane (*hom*) family were detected in *H. suis*. Besides the major *H. pylori *OMP family proteins, the *H. suis *genome contains some predicted OMPs based on their N-terminal pattern of alternating hydrophobic amino acids similar to porins, encompassing *omp29 *for HS1^T ^and *omp11 *and *omp29 *for HS5. A 491 amino acids membrane-associated homolog of the virulence factor MviN, aligned for 92% with the MviN homolog of *H. acinonychis *(Hac_1250), was also present in *H. suis*.

### Type IV secretion systems in *H. suis*

Of the *H. pylori *type IV secretion systems (T4SS), only two members of the *cag *pathogenicity island (*cag*PAI) were identified in the *H. suis *genome (*cag23/E *and *cagX*). Most members of the *comB *transport apparatus were present. These include *comB2*, *B3*, *B6*, *B8 *and a number of additional genes not classified as *comB*: *recA*, *comE*, *comL *and *dprA*. *H. suis *possesses genes encoding VirB- and VirD-type ATPases (*virB4*, *B8*, *B9*, *B10*, *B11*, and *virD2*, *D4*), all designated members of the *H. pylori *type IV secretion system 3 (*tfs3*). The HS1^T ^and HS5 T4SS are presented in Table 2.

**Table 2:** *H. suis *strain 1 (HS1^T^) and strain 5 (HS5) homologs of *H. pylori *and other *Helicobacter sp*. type IV secretion system genes.

Homolog	Gene detected in HS1^T^	Gene detected in HS5	Description of corresponding protein	Percentage of sequence fraction aligned (of which % conserved) with *Helicobacter *homolog^1^
***cag *pathogenicity island**				
*cag23*/*E *of *H. pylori*	HSUHS1_0731	HSUHS5_1234	DNA transfer protein	81 (42)
*cagX *of *H. pylori*	HSUHS1_0964	HSUHS5_0688	Conjugal plasmid transfer protein	92 (71)

***comB *system**				
*comB2 *of *H. acinonychis*	HSUHS1_1181	HSUHS5_0010	ComB2 protein	96 (64)
*comB3 *of *H. acinonychis*	HSUHS1_1182	HSUHS5_0009	ComB3 competence protein	95 (77)
*comB6 *of *H. pylori*	HSUHS1_0337	-	NADH-ubiquinone oxidoreductase	70 (85)
*comB8 *of *H. pylori*	HSUHS1_0747	Overlap with *virB8*	comB8 competence protein	93 (66)
*trbL *of *H. pylori*	HSUHS1_0755	HSUHS5_0054	TrbL protein	99 (77)
*comE *of *H. acinonychis*	HSUHS1_0314	HSUHS5_0381	Competence locus E	94 (55)
*comL *of *H. pylori*	HSUHS1_0722	HSUHS5_0300	Competence protein	99 (84)
*dprA *of *H. acinonychis*	HSUHS1_0096	HSUHS5_0824	DNA processing protein	99 (70)
*recA *of *H. hepaticus*	HSUHS1_0672	HSUHS5_1058	Recombinase A	97 (84)

***virB *-homologs**				
*virB4 *of *H. pylori*	HSUHS1_0960	HSUHS5_0692	DNA transfer protein	98 (68)
*virB8 *of *H. pylori*	HSUHS1_0963	HSUHS5_0689	DNA transfer protein	91 (61)
*virB9 *of *H. cetorum*	HSUHS1_0319	-	VirB9 protein	76 (69)
*virB10 *of *H. cetorum*	HSUHS1_0320	-	VirB10 protein	90 (77)
putative *virB9 *of *H. pylori*	-	HSUHS5_0372	Putative VirB9 protein	100 (86)
putative *virB10 *of *H. pylori*	-	HSUHS5_0371	Putative VirB10 protein	97 (87)
*virB11 *of *H. pylori*	HSUHS1_0750	HSUHS5_0368	VirB11 protein	100 (98)
*virB11 *of *H. cetorum*	HSUHS1_0965	-	VirB11 protein	95 (71)
*virB11*-like *of H. pylori *(HPSH_04565)	-	HSUHS5_0686	VirB11-like protein	98 (72)
*virB11*-like *of H. pylori *(HPSH_07250)	HSUHS1_0036	HSUHS5_0600	Type IV ATPase	100 (75)

***virD *- homologs**				
*virD2 *of *H. cetorum*	HSUHS1_0752	HSUHS5_0414	VirD2 protein (relaxase)	100 (90)
*virD4 *of *H.pylori*	HSUHS1_0870	HSUHS5_0257	VirD4 protein (conjugation protein)	82 (78)

^1 ^Resulting from tblastn-based cross-mapping of the *H. pylori *proteome to the *H. suis *HS1^T ^and HS5 genomes and blastp-based *ab initio *analyses of the translated *H. suis *HS1^T ^and HS5 ORFs against the Uniprot-KB universal protein database. Differences between HS1^T ^and HS5 homologs ≤ 1%.

### Genes possibly involved in induction of gastric lesions

Homologs of *H. pylori *genes involved in induction of gastric lesions in the *H. suis *genome are summarized in Table 3. Homology searches with the *H. pylori *vacuolating cytotoxin A gene (*vacA*) identified HSUHS1_0989 in HS1^T^. The corresponding protein, which is exceptional in that it is one of the longest in the world of prokaryotes, possesses three small conserved VacA regions (residues 490-545, 941-995, and 1043-1351), followed by an autotransporter region (residues 2730-2983). The amino acid sequence of the HS5 homolog (HSUHS5_0761) could be aligned for 22% with the *H. pylori *strain HPAG1 sequence, and possesses only one conserved VacA region (residues 242-298), followed by an autotransporter region (1258-1510). In both *vacA *homologs, no signal sequence was determined. Additionally, an ulcer-associated adenine-specific DNA methyltransferase (HSUHS1_0375, HSUHS5_0957) coding sequence was identified, whereas a molecular homolog of the ulcer-associated restriction endonuclease (*iceA*) could not be discovered in *H. suis*. *H. suis *contains homologs of *pgbA *and *pgbB *encoding plasminogen-binding proteins, though both lacking a transmembrane helix or signal peptide cleavage site according to the bioinformatics tools mentioned earlier. *H. suis *harbours homologs of genes coding for the *H. pylori *neutrophil-activating protein (HP-NapA) and γ-glutamyl transpeptidase (HP-GGT). Homologs encoding the *H. pylori *flavodoxin *fldA *and the pyruvate-oxidoreductase complex (POR) members *porA*, *porB*, *porC*, and *porD *were also identified in *H. suis*.

**Table 3:** Homologs of *H. pylori *genes involved in induction of gastric lesions in the *H. suis *type strain 1 (HS1^T^) and strain 5 (HS5) genome.

Gene detected in HS1^T^	Gene detected in HS5	Gene name	Protein annotation/function in *H. pylori*	Sequence fraction HS1^T^/HS5 aligned with *H. pylori *homolog (%)^1^	Aligned sequence fraction HS1^T^/HS5 conserved with *H. pylori *homolog (%)^1^	References
HSUHS1_0989	HSUHS5_0761	*vacA*	Vacuolating cytotoxin A: host cell vacuolation, apoptosis-inducing, immunosuppresive	63/22	45/72	[46]
HSUHS1_0265	HSUHS5_0449	*ggt*	γ-glutamyl transpeptidase: apoptosis-inducing, immunosuppresive	99/99	86/86	[48,49,64]
HSUHS1_1177	HSUHS5_0014	*napA*	Neutrophil-activating protein A: proinflammatory	99/99	83/83	[50,51]
HSUHS1_1067	HSUHS5_1177	*fldA*	Electron acceptor of the pyruvate oxidoreductase enzyme complex, associated with gastric MALT lymphoma in humans	96/98	84/83	[55,56]
HSUHS1_0403	HSUHS5_0887	*pgbA*	Plasminogen-binding protein	60/60	72/72	[53,54]
HSUHS1_1192	HSUHS5_0523	*pgbB*	Plasminogen-binding protein	70/70	72/72	[53,54]

^1^Resulting from tblastn-based cross-mapping of the *H. pylori *proteome to the *H. suis *HS1^T ^and HS5 genomes.

## Discussion

### Genes possibly involved in gastric colonization and persistence

The results of the present study demonstrate that several *H. pylori *genes involved in acid acclimation, chemotaxis and motility, have counterparts in the *H. suis *genome. These genes are known to be essential for colonization of the human gastric mucosa [27-32].

Several OMP coding sequences were identified by comparative analyses with *H. pylori *and other bacterial species. *H. suis *contains some similar members of the major OMP families described in *H. pylori *[33]. Some of these OMPs have been described to be involved in adhesion of *H. pylori *to the gastric mucosa, which is widely assumed to play an important role in the initial colonization and long-term persistence in the human stomach. These include the gastric epithelial cell adhesin HorB [34] and the surface lipoprotein, *H. pylori *adhesin A (HpaA). HpaA, also annotated as neuraminyllactose-binding hemagglutinin, is found exclusively in *Helicobacter *and binds to sialic acid-rich macromolecules present on the gastric epithelium [35]. On the other hand, *H. suis *lacks homologs of several other *H. pylori *adhesion factors, including genes coding for the blood group antigen binding adhesins *babA *(*hopS*) and *babB *(*hopT*), the sialic acid binding adhesins *sabA *(*hopP*) and *sabB *(*hopO*), and the adherence-associated lipoproteins *alpA *(*hopC*) and *alpB *(*hopB*) [36].

*H. suis *contains a fibrinonectin/fibrinogen-binding protein coding gene, which may enhance its adherence to injured gastric tissue. Damage to host epithelial cells may indeed expose fibronectin and other extracellular matrix components. Strong homology was found with fibronectin-binding proteins of *H. felis *(YP_004072974), *H. canadensis *(ZP_048703091) and *Wolinella succinogenes *(NP_907753). To our knowledge, no exact function has been given to these proteins in these species. In *Campylobacter jejuni*, however, fibronectin-binding proteins CadF and FlpA have been shown to be involved in adherence to and/or invasion of host's intestinal epithelial cells [37,38]. According to the bioinformatics tools used here, the *H. suis *fibronectin-binding protein lacks a transmembrane helix or signal peptidase cleavage site, indicating that it is not surface exposed or secreted. Its real role in colonization therefore remains to be elucidated.

Three genes involved in sialic acid biosynthesis (*neuA*, *neuB*, and *wecB*) were annotated in the *H. suis *genome, indicating that this bacterium may decorate its surface with sialic acid. The presence of surface sialylation has been studied extensively in pathogenic bacteria, where it contributes to evasion of the host complement defense system [39].

Additionally, *H. suis *possesses genes encoding enzymes involved in oxidative-stress resistance (*napA*, *sodB*, *katA*, *mutS*, *mdaB*, and peroxiredoxin coding sequence). This indicates that *H. suis *may harbour a defense mechanism against the host inflammatory response, contributing to the ability of chronic gastric colonization by this bacterium [40].

### Type IV secretion systems in *H. suis*

Two partial T4SS were predicted in the *H. suis *genome, namely the *comB *cluster and the *tfs3 *system. The *H. suis comB *system probably plays a role in genetic transformation [41,42]. Transformation of DNA can be responsible for the high degree of diversity among *H. suis *strains as has been recently demonstrated by multilocus sequence typing of available *H. suis *strains [43]. The role of the *H. pylori tfs3 *secretion system in pathogenesis is not exactly known. Seven genes of the *tfs3 *cluster are homologs of genes involved in type IV secretion: *virB4*, *virB11*, and *virD4 *code for ATPases which move substrates to and through the pore. The latter is coded by transmembrane pore genes *virB7*, *virB8*, *virB9*, and *virB10 *[44]. All these genes, except *virB7 *were identified in *H. suis*, indicating that the *H. suis tfs3 *can be important in transmembrane transport of substrates in *H. suis*.

The *H. pylori cag *pathogenicity island (*cag*PAI) region encodes a T4SS allowing *H. pylori *to insert the cytotoxin-associated antigen A (CagA) into the host cell. This process results in altered host cell structure, an increased inflammatory response, and a higher risk for gastric adenocarcinoma [45]. Although *H. suis *possesses two members of the *H. pylori cag*PAI (*cag23/E *and *cagX*), the majority of genes, including the gene coding for pathology-causing protein (CagA), were not identified. This indicates that HS1^T ^and HS5 lack a functional *cag *protein transporter secretion system.

### Genes possibly involved in induction of gastric lesions

Genomic comparison of *H. suis *with *H. pylori *resulted in the identification of additional genes possibly associated with virulence in *H. suis*. A *H. suis *homolog of the *H. pylori vacA *was detected. VacA is both a cytotoxin of the gastric epithelial cell layer, and an immunomodulatory toxin of *H. pylori *[46]. *H. pylori *contains either a functional or non-functional *vacA*. The *H. suis vacA *homolog exhibits no *vacA *signal sequence, indicating that it might encode a non-functional cytotoxin [47]. In vitro and in vivo studies with a knockout mutant of the *H. suis vacA *could clarify the functionality of the *vacA *homolog in this *Helicobacter *species.

Strong homology was found with two *H. pylori *virulence-associated genes namely *napA*, encoding the HP-NapA and *ggt*, encoding HP-GGT. The *H. pylori *GGT has been identified as an apoptosis-inducing protein [48,49]. The HP-NapA protein is designated as a proinflammatory and immunodominant protein by stimulating production of oxygen radicals and IL-12 from neutrophils and recruiting leukocytes in vivo [50,51]. Moreover, HP-NapA also plays a role in protecting *H. pylori *from oxidative stress by binding free iron [52]. *H. suis *contains homologs of two *H. pylori *genes coding for plasminogen-binding proteins, *pgbA *and *pgbB*. The corresponding proteins, PgbA and PgbB bind host plasminogen, which subsequently can be activated to plasmin and may contribute to obstructing the natural healing process of gastric ulcers [53,54]. The biological role of the *H. suis pgbA *and *B *homologs in chronicity of gastric ulceration is uncertain, as no exact membrane association was found in the corresponding proteins.

The risk to develop MALT lymphomas in *H. suis *infected human patients is higher than after infection with *H. pylori *[5,14]. Homologs encoding the *H. pylori *flavodoxin (*fldA*) and its electron donor, the POR enzyme complex (*porA *to *D*) were found in *H. suis*. The *H. pylori *flavodoxin protein (FldA) has been proposed to play a role in the pathogenesis of *H. pylori*-associated MALT lymphoma, as antibodies against the *H. pylori *FldA protein were more prevalent in patients with MALT lymphomas compared to patients with other *H. pylori-*related diseases [55]. Besides, insertion mutagenesis of the *fldA *and the *por *complex has shown that these genes are essential for the survival of *H. pylori *[56]. These observations indicate that *fldA *and its *por *complex may play a role in gastric colonization of *H. suis *and MALT lymphoma development in *H. suis *infected people.

Recently, the genomes of the carcinogenic *H. pylori *strain B38 and the carcinogenic and ulcerogenic *Helicobacter mustelae *have been sequenced [57,58]. Both helicobacters lack homologs of major *H. pylori *virulence genes (e.g. *cagA*, *babA*/*B*, *sabA*/*B*), which are also absent in the *H. suis *genome. Additionally, *H. mustelae *lacks a *vacA *homolog. Despite this absence, infection with *H. pylori strain *B38 and *H. mustelae *has been associated with gastric MALT lymphomas and other gastric disorders. Whole genome sequencing data are also available from *H. acinonychis *strain Sheeba, a gastric pathogen of large felines. Similar to *H. suis*, *H. acinonychis *lacks a *cag*PAI as well as genes encoding BabA/B and SabA/B. Both species contain a *vacA *homolog, which for *H. acinonychis *has been described to be fragmented [59,60].

*H. suis *contains a *mviN *homolog. This gene has been described to be a virulence factor of several bacterial species, such as *Burkholderia pseudomallei *and *Vibrio alginolyticus *[61,62]. In addition to virulence, MviN has been described to be essential for in vitro growth of these and other bacteria [61-63]. The biological significance of *mviN *in the *Helicobacter *genus, however, remains to be elucidated.

## Conclusion

Although *H. suis *lacks homologs of some major *H. pylori *virulence genes, other candidate virulence factors, such as *napA*, *ggt*, *mviN*, and *fldA *were detected. *H. suis *also possesses genes known to be essential for gastric colonization. Future in vitro and in vivo research of the currently presented genes of this porcine and human gastric pathogen should elucidate their precise role in colonization and virulence.

### Nucleotide sequence accession numbers

The genome sequences have been deposited at GenBank/EMBL/DDBJ under the accession ADGY00000000 for HS1^T ^and ADHO00000000 for HS5. The versions described in this paper are the first versions, ADGY1000000 and ADHO1000000.

## Competing interests

The authors declare that they have no competing interests.

## Authors' contributions

MV designed the study, analysed the data and drafted the manuscript. TTMV carried out the sequence alignment and participated in the design of the study. BF, AS and DDG participated in the design of the study. FP, WVC, RD, and FH coordinated and participated in the design of the study. All authors read and approved the final manuscript.

## Supplementary Material

Additional file 1 **Table S1 Classification of *H. suis *strain 1 (HS1**^**T**^**) and strain 5 (HS5) outer membrane proteins (OMPs) in relation to *H. pylori *OMPs**. Additional file Table S1 presents the classification of *H. suis *outer membrane proteins in relation to *H. pylori *outer membrane proteins. Although this table is not essential, we believe that it is both a relevant and interesting addition to the content of the article. Click here for file
