# Supplementary material for: Genome sequence of Helicobacter suis supports its role in gastric pathology
Source: Vet Res. 2011 Mar 17;42(1):51. doi: 10.1186/1297-9716-42-51 (PMC3065412; doi:10.1186/1297-9716-42-51)
Supplement: Additional file 1 — Table S1 Classification of H. suis strain 1 (HS1T) and strain 5 (HS5) outer membrane proteins (OMPs) in relation to H. pylori OMPs. Additional file Table S1 presents the classification of H. suis outer membrane proteins in relation to H. pylori outer membrane proteins. Although this table is not essential, we believe that it is both a relevant and interesting addition to the content of the article. [file 1297-9716-42-51-S1.DOC]

Additional file 1

**Table S1.** Classification of *H. suis* strain 1 (HS1^T^) and strain 5 (HS5) outer membrane proteins (OMPs) in relation to *H. pylori* OMPs.

| **OMP family** | **Gene detected in HS1^T^** | **Gene detected in HS5** | **Percentage of sequence fraction aligned (of which % conserved) with *H.pylori* homolog^1^** |
| --- | --- | --- | --- |
| 1. **Hop-related OMPs**   HopE  HopG-2  HopH^2^  HopW protein precursor | HSUHS1_0015  HSUHS1_0180  HSUHS1_0340  HSUHS1_1105 | HSUHS5_0838  HSUHS5_1092  HSUHS5_0513  - | 99 (57)  92 (79)  83 (49)  90 (71) |
| 1. **Hor-related OMPs**   HorA  HorB  HorC  HorD  HorE  HorF  HorJ  HorL | -  HSUHS1_0666  HSUHS1_0472  HSUHS1_0202  HSUHS1_1144  -  HSUHS1_0188  - | HSUHS5_1252  HSUHS5_1053  HSUHS5_0483  HSUHS5_0115  -  HSUHS5_0133  HSUHS5_0782  HSUHS5_0611 | 100 (52)  100 (63)  99 (63)  91 (77)  96 (64)  85 (76)  99 (53)  93 (67) |
| 1. **Hof-related OMPs**   HofA  HofC  HofE  HofF  Hof-family OMP | HSUHS1_0120  HSUHS1_0181  HSUHS1_0179  HSUHS1_0178  HSUHS1_0182 | HSUHS5_0671  HSUHS5_1091  HSUHS5_1093  HSUHS5_1094  HSUHS5_1090 | 93 (74)  99 (73)  98 (61)  95 (85)  95 (84) |
| 1. **Iron-regulated OMPs**   Iron(III) dicitrate transport protein (FecA)  Iron(III) dicitrate transport protein (FecA)  Iron-regulated OMP (FrpB) | HSUHS1_0092  HSUHS1_1124  HSUHS1_1106 | HSUHS5_0819  HSUHS5_1010  HSUHS5_0783 | 100 (89)  96 (76)  99 (79) |
| 1. **Efflux pump OMPs**   Omp of the hefABC efflux system (HefA)  Membrane fusion protein of the hefABC efflux system (HefB)  Cytoplasmic pump protein of the hefABC efflux system (HefC) | HSUHS1_0643  HSUHS1_0644  HSUHS1_0645 | HSUHS5_1029  HSUHS5_1030  HSUHS5_1031 | 82 (77)  92 (79)  100 (78) |
| 1. **Unclassified OMPs**^3^   Omp11 of *H. pylori*  Omp29 of *H. pylori*  Putative Omp of *H. pylori* (HPSH_03820)  Putative Omp of *H. pylori* (HP_1525)  Putative Omp of *H.pylori* (HPSH_03230)  Putative Omp of *H. pylori* (jhp_0368)  Putative Omp of *H. pylori* (jhp_0370)  Putative Omp of *H. pylori* (HP_1056)  Putative Omp of *H. pylori* (HPSH_02040)  Putative Omp of *H. pylori* (HPSH_02045) | -  HSUHS1_0008  HSUHS1_0646  HSUHS1_1135  HSUHS1_0446  HSUHS1_0585  HSUHS1_0583  HSUHS1_0584  -  - | HSUHS5_1235  HSUHS5_1251  HSUHS5_1032  HSUHS5_0620  HSUHS5_0930  -  -  -  HSUHS5_0158  HSUHS5_0156 | 99 (68)  94 (46)  100 (58)  94 (72)  96 (56)  100 (67)  95 (84)  74 (69)  75 (66)  95 (59) |

^1^ Resulting from tblastn-based cross-mapping of the *H. pylori* proteome to the *H. suis* HS1^T^ and HS5 genomes and blastp-based *ab initio* analyses of the translated *H. suis* HS1^T^ and HS5 ORFs against the Uniprot-KB universal protein database. Differences between HS1^T^ and HS5 homologs ≤ 1%.

^2^ 99 amino acid HopH of *H. pylori* strain Shi 470 (HPSH_03675).

^3^ Predicted OMPs based on their N-terminal pattern of alternating hydrophobic amino acids similar to porins.
